# Supplementary material for: Antimicrobial Peptide Arsenal Predicted from the Venom Gland Transcriptome of the Tropical Trap-Jaw Ant Odontomachus chelifer
Source: Toxins (Basel). 2023 May 18;15(5):345. doi: 10.3390/toxins15050345 (PMC10221683; doi:10.3390/toxins15050345)
Supplement: Supplementary file 1 [file toxins-15-00345-s001.zip › toxins-2327919-supplementary.pdf]

Supplementary Materials: Antimicrobial Peptide Arsenal Predicted from the Venom Gland Transcriptome of the Tropical Trap-Jaw Ant *Odontomachus chelifer*

Josilene J. Menk, Yan E. Matuhara, Henrique Sebestyen-França, Flávio Henrique-Silva, Milene Ferro, Renata Santos Rodrigues and Célio D. Santos-Júnior

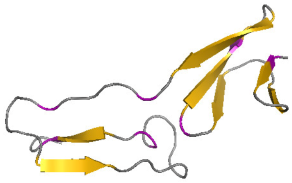

Figure S1. Tridimensional structure prediction with marked cysteine residues of the putative poneratoxin ICK-like toxin homolog of *O. chelifer* (TRINITY_DN24839_c0_g1_i1.p1). Yellow: strand. Gray: turns. Purple: cysteine residues.

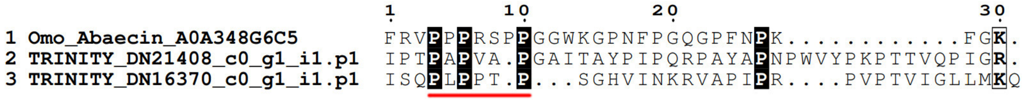

Figure S2. Primary sequence alignment between two predicted AMP sequences from *O. chelifer* and the A0A348G6C5 abaecin from *O. monticola*. Similar residues are boxed. Identical residues are bolded. The red line indicates the PXP/PXXP motifs.

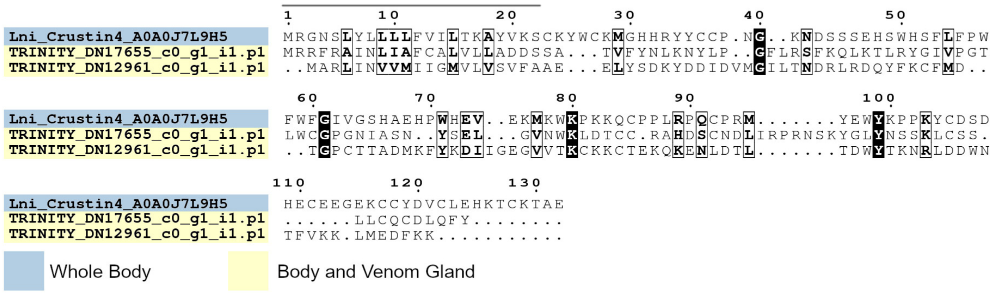

Figure S3. Global alignment between the reference crustin and the grouped candidate AMP sequences. The color specifies the source tissue. The gray line indicates the signal peptide of the reference AMP.

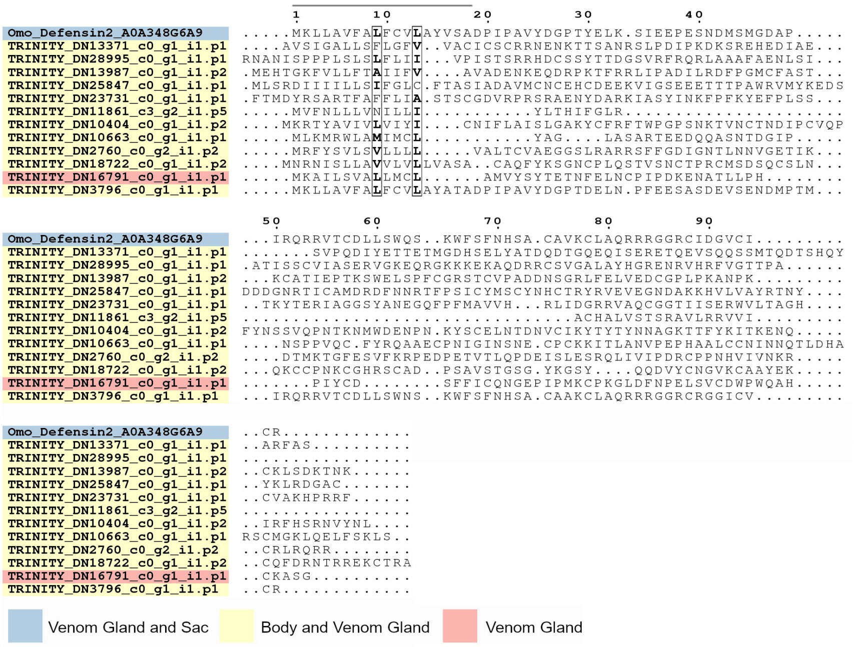

Figure S4. Global alignment between the reference defensin and the grouped candidate AMP sequences. The color specifies the source tissue. The gray line indicates the signal peptide of the reference AMP.

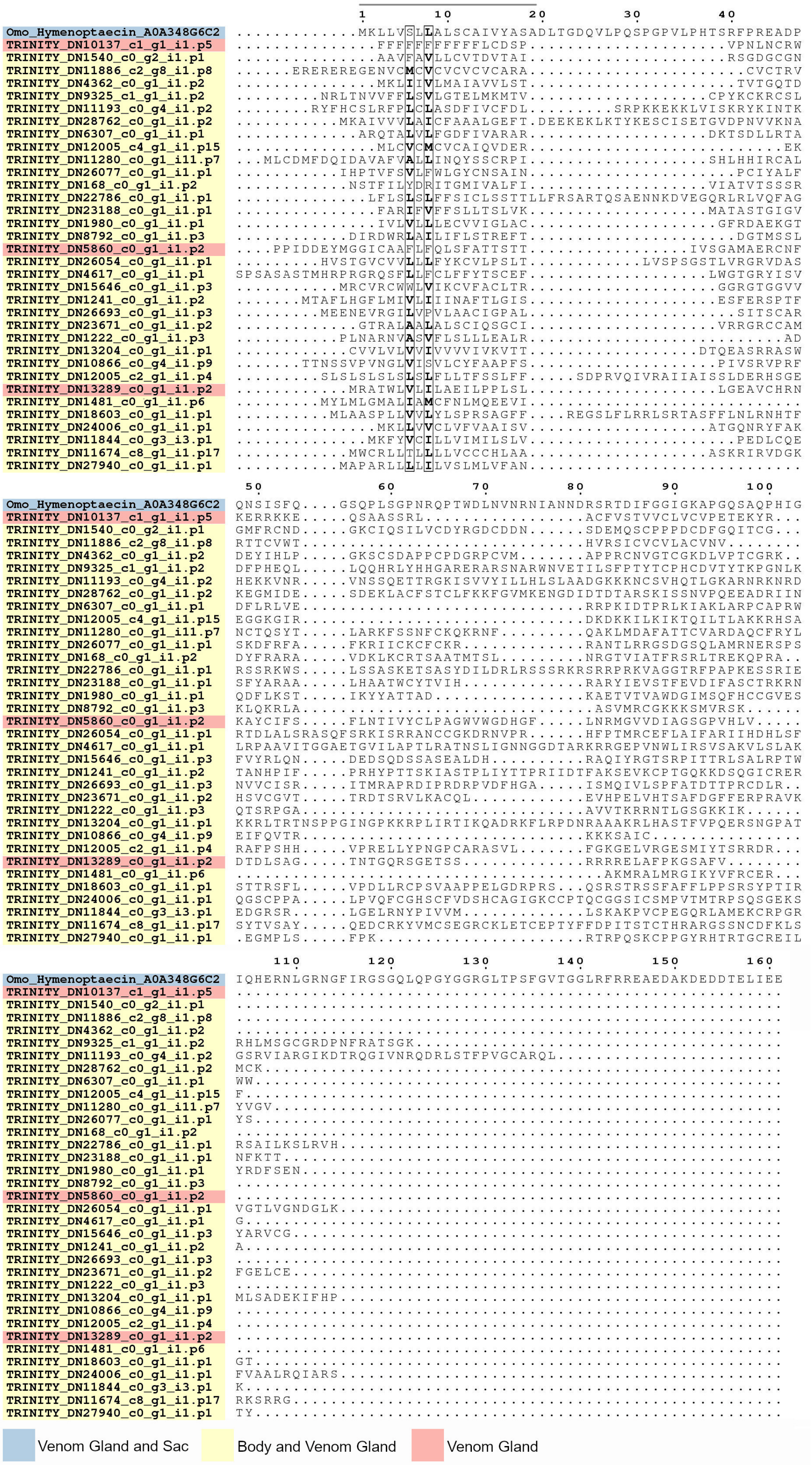
Omo_Hymenoptaecin_A0A348G6C2
TRINITY_DN10137_c1_g1_i1.p5
TRINITY_DN1540_c0_g2_i1.p1
TRINITY_DN11886_c2_g8_i1.p8
TRINITY_DN4362_c0_g1_i1.p2
TRINITY_DN9325_c1_g1_i1.p2
TRINITY_DN11193_c0_g4_i1.p2
TRINITY_DN28762_c0_g1_i1.p2
TRINITY_DN6307_c0_g1_i1.p1
TRINITY_DN12005_c4_g1_i1.p15
TRINITY_DN11280_c0_g1_i11.p7
TRINITY_DN26077_c0_g1_i1.p1
TRINITY_DN168_c0_g1_i1.p2
TRINITY_DN22786_c0_g1_i1.p1
TRINITY_DN23188_c0_g1_i1.p1
TRINITY_DN1980_c0_g1_i1.p1
TRINITY_DN8792_c0_g1_i1.p3
TRINITY_DN5860_c0_g1_i1.p2
TRINITY_DN26054_c0_g1_i1.p1
TRINITY_DN4617_c0_g1_i1.p1
TRINITY_DN15646_c0_g1_i1.p3
TRINITY_DN1241_c0_g1_i1.p2
TRINITY_DN26693_c0_g1_i1.p3
TRINITY_DN23671_c0_g1_i1.p2
TRINITY_DN1222_c0_g1_i1.p3
TRINITY_DN13204_c0_g1_i1.p1
TRINITY_DN10866_c0_g4_i1.p9
TRINITY_DN12005_c2_g1_i1.p4
TRINITY_DN13289_c0_g1_i1.p2
TRINITY_DN1481_c0_g1_i1.p6
TRINITY_DN18603_c0_g1_i1.p1
TRINITY_DN24006_c0_g1_i1.p1
TRINITY_DN11844_c0_g3_i3.p1
TRINITY_DN11674_c8_g1_i1.p17
TRINITY_DN27940_c0_g1_i1.p1
Venom Gland and Sac
Body and Venom Gland
Venom Gland

Figure S5. Global alignment between the reference hymenoptaecin and the grouped candidate AMP sequences. The color specifies the source tissue. The gray line indicates the signal peptide of the reference AMP.

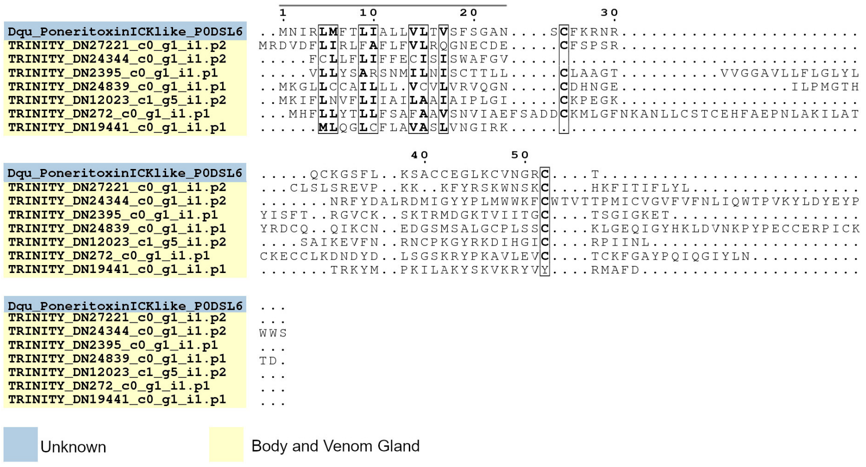

Figure S6. Global alignment between the reference ICK-type AMP and the grouped candidate AMP sequences. The color specifies the source tissue. The gray line indicates the signal peptide of the reference AMP.

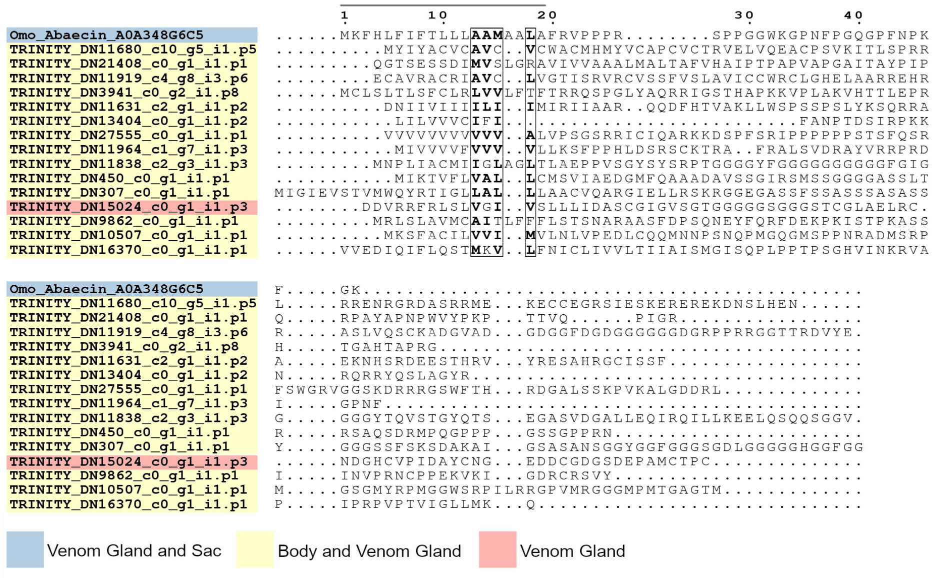

Figure S7. Global alignment between the reference abaecin and the grouped candidate AMP sequences. The color specifies the source tissue. The gray line indicates the signal peptide of the reference AMP.
